# The Preparation and Crystal Structures of Octaoxoketocalix[8]arene Derivatives: The Ketocalixarene Counterparts of the Largest “Major” Calixarene

**DOI:** 10.3390/molecules29174094

**Published:** 2024-08-29

**Authors:** Katerina Kogan, Suheir Omar, Benny Bogoslavsky, Silvio E. Biali

**Affiliations:** Institute of Chemistry, The Hebrew University of Jerusalem, Jerusalem 9190401, Israel; kogan.katerina@gmail.com (K.K.); suheir.omar@mail.huji.ac.il (S.O.); bennybo@savion.huji.ac.il (B.B.)

**Keywords:** calixarenes, oxidation, macrocycles

## Abstract

The purpose of this study was to synthesize and structurally characterize ketocalixarenes (i.e., calixarenes where the bridging methylene bridges are replaced by carbonyl groups) derived from the largest “major” calixarene, namely *p*-*tert*-butylcalix[8]arene **3a**. Ketocalix[8]arenes were synthesized by the oxidation of protected *p*-*tert*-butylcalix[8]arene derivatives. Octamethoxy-p-tert-butylketocalix[8]arene **6b** was prepared by the photochemical reaction of the calixarene **3b** with NBS in a CHCl_3_/H_2_O mixture. The oxidation of the methylene groups of octaacetoxy-*p*-*tert*-butylcalix[8]arene **3c** was conducted by a reaction with CrO_3_ in Ac_2_O/AcOH. The basic hydrolysis of the acetate groups of the oxidation product yielded octahydroxy-*p*-*tert*-butylketocalix[8]arene **6a**. In the crystal, the molecule adopts a saddle-like conformation of crystallographic *C*_2_ and idealized *S*_4_ symmetry. Strikingly, the array of OH/OH intramolecular hydrogen bonds present in the parent **3a** is completely disrupted in **6a**.

## 1. Introduction

**Scheme 1:**
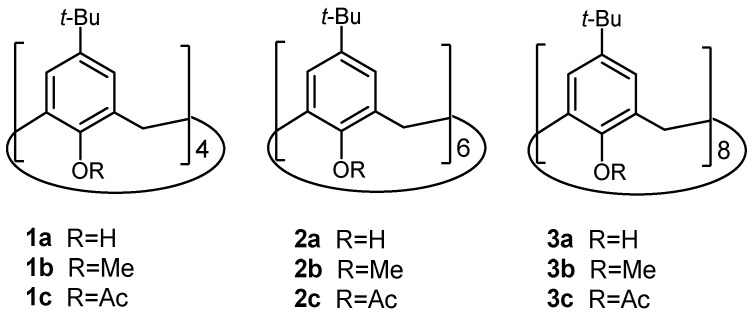
“Major” calixarenes **1a**–**3a** and their protected derivatives.

Calix[*n*]arenes are synthetic macrocycles consisting of *n* phenol rings interconnected by methylene units [1,2,3,4,5,6]. These systems possess a well-defined cavity capable of hosting smaller molecules or ions. During the last few decades, calixarenes have received considerable attention because of their multitude of potential applications [6]. Their preparation usually involves the base-catalyzed condensation of *p*-*tert*-butylphenol and formaldehyde. Only three calixarenes were initially synthetically accessible in both multigram scale and high yields: *p*-*tert*-butylcalix[4]arene (**1a**), *p*-*tert*-butylcalix[6]arene (**2a**) and *p*-*tert*-butylcalix[8]arene (**3a**) [7,8,9,10]. These systems were dubbed by Gutsche as “major calixarenes”(Scheme 1) [11]. Substituents are usually introduced at the scaffold via the electrophilic substitution of aromatic rings and/or via the alkylation or acylation of phenolic hydroxyl groups [12]. These modifications usually aim to alter the chemical properties, three-dimensional shape and rigidity of the macrocycle. A structural modification which is relatively unexplored involves the oxidation of methylene bridges to carbonyl groups.

**Figure 1:**
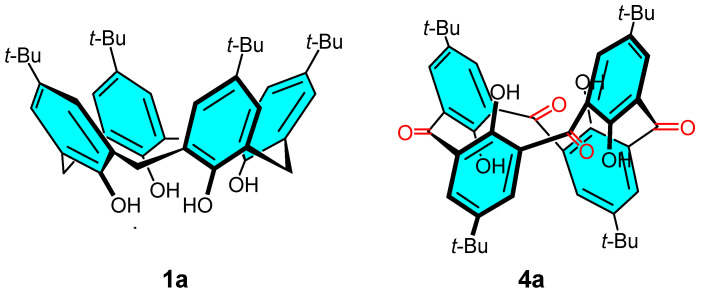
Tetrahydroxy-*p*-*tert*-butyl-calix[4]arene **1a** (**left**) adopts a *cone* conformation, while tetrahydroxyketocalix[4]arene **4a** (**right**) adopts a 1,3-*alternate* conformation.

Calixarenes where phenol rings are interconnected by carbonyl groups (“ketocalixarenes”) are of both stereochemical and synthetic interest [13]. The formal replacement of the bridging methylene groups by carbonyls changes the conformational preferences of the macrocycle. Remarkably, whereas the parent *p*-*tert*-butylcalix[4]arene (**1a**) adopts a *cone* conformation stabilized by a circular array of hydrogen bonds [14], tetrahydroxyketocalix[4]arene **4a** adopts in the crystal and in solution a *1*,*3*-*alternate* conformation (Figure 1) [15,16]. The carbonyl groups are not involved in intramolecular hydrogen bonding, and it was conjectured that the conformational change is due to dipole–dipole repulsion in the *cone* form and the increased conjugation of aryl rings with carbonyl groups in the conformation adopted [15].

**Scheme 2:**
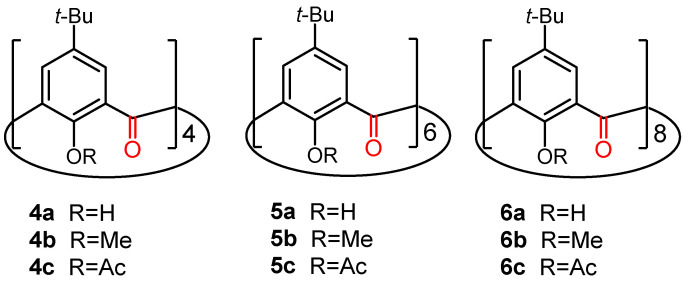
Ketocalixarene derivatives of “major” calixarenes.

Tetrahydroxyketocalix[4]arene **4a** (Scheme 2) was first synthesized in 1990 by Görmar and coworkers [17]. The reaction sequence involved the acetylation of the hydroxyl groups of **1a** (to protect the phenol moieties during the subsequent oxidation step), CrO_3_ oxidation of the methylene groups of **1c** and hydrolysis of the acetoxy groups [17,18]. Starting from hexahydroxycalix[6]arene **2a**, a similar synthetic sequence was utilized for the preparation of hexahydroxyketocalix[6]arene **5a** [19]. There is only a single report on the attempted oxidation of the methylenes of a derivative of calix[8]arene **3a** which is the largest “classical” calixarene. In a pioneering study, Ninagawa and coworkers reported in 1985 on the CrO_3_ oxidation of the methylene groups of **3c** in an Ac_2_O/AcOH mixture at 45 °C. A calix[8]arene derivative was obtained with three methylene bridges oxidized to carbonyls, but the identity of the structural isomer isolated was not determined [20]. The goal of this work was to synthesize and structurally characterize the derivatives of *p*-*tert*-butylcalix[8]arene, where all methylene bridges were oxidized to carbonyl groups.

## 2. Results

### 2.1. General Considerations

In all cases reported where the methylene groups of a calix scaffold were oxidized to carbonyl groups, the calixarene phenolic OH groups were protected as methyl ether (i.e., **1b**, **2b**) or acetoxy (**1c**, **2c**) groups. This protection is necessary to avoid the oxidation of the phenol rings (e.g., to yield calixquinones [21] or 5,5’-bicalix[*n*]arenes [22]). In general, methyl ethers functionalities are considered more robust protecting groups of phenolic hydroxyls. This is of importance if, after oxidation, a further chemical modification of carbonyl bridges is planned on the protected derivative (e.g., by reaction with an organometallic reagent). For example, the reaction of tetramethoxyketocalix[4]arene **4b** with PhLi [23] or MeLi [24] proceeds, readily affording the corresponding tetra-addition product. However, in our previous experience, we found that the deprotection of all the OH groups by the cleavage of the O-Me bonds in a methylene functionalized system sometimes fails, even under strenuous reaction conditions. Labile acetoxy protecting groups, although incompatible with organometallic reagents, can be readily cleaved under basic hydrolysis conditions and unmask protected OH groups.

### 2.2. Oxidation of Octamethylether of Calix[8]arene

**Figure 2:**
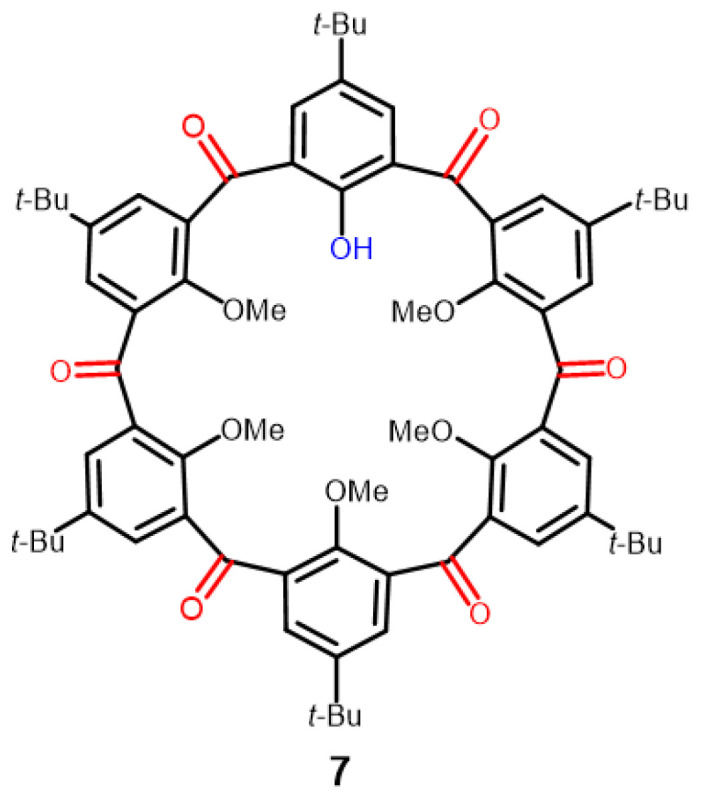
Side product **7** obtained in the oxidation of **2b**.

In 2013, Fischer and coworkers reported on a facile method for the oxidation of the methylene groups of tetramethoxycalix[4]arene **1b**. The photochemical reaction of **1b** with eight equivalents of N-bromosuccinimide (NBS) in a mixture of CHCl_3_/H_2_O afforded ketocalix[4]arenes **4b** in relatively high yields [25,26]. Presumably, the reaction involved the radical dibromination of methylenes, followed by the hydrolysis of the brominated bridges. In the case of the larger **2b**, the initial experiments showed that the reaction afforded the target **5b**, but a pentamethoxy ketocalix[6]arene derivative (**7**, Figure 2) was also obtained as a side product, presumably formed via the cleavage of an O-Me bond by the HBr generated in the reaction. The optimization of the reaction time yielded the ketocalixarene **5b** in high yield [27].

The oxidation of **3b** (NBS, irradiation with a spot lamp, CHCl_3_/H_2_O) was conducted using the optimized reaction conditions found previously for **2b** (Equation (1)). An examination of the crude product by NMR indicated the formation of a single major product. After recrystallization from CHCl_3_/EtOH, **6b** was isolated in 36% yield.
(1)
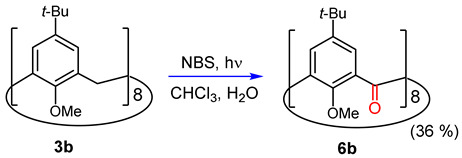


The **^1^**H NMR spectrum of **6b** (500 MHz, CDCl_3_, rt) displayed a symmetrical pattern, with one singlet each for the aromatic, methoxy and *t*-Bu protons (Supplementary Materials). The observed downfield shift in the aromatic protons (δ = 7.60 ppm) is consistent with the presence of carbonyl groups at the bridges, which results in the deshielding of these protons. A symmetrical pattern was also observed in the ^13^C NMR spectrum. Both NMR spectra are consistent with a molecule possessing averaged eightfold symmetry.

### 2.3. Crystal Structure of Ketocalix[8]arene ***6b***

**Figure 3:**
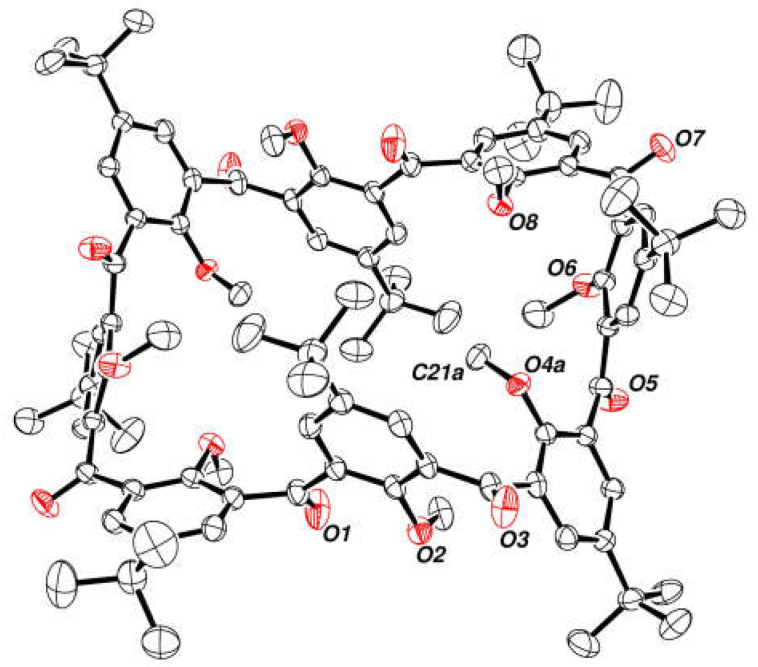
The crystal structure of octamethoxyketocalix[8]arene **6b**. Chloroform molecules that cocrystallized are omitted. A pair of opposite methoxy groups and *t*-Bu groups are disordered. For clarity, only one orientation of the disordered groups is shown.

Single crystals of **6b** were grown from a mixture of CHCl_3_/EtOH. The molecule crystallized with CHCl_3_ molecules (Figure 3) in a conformation of *C*_i_ symmetry. A pair of opposite methoxy groups (C(21a)-O(4a) and C(21b)-O(4b)) were disordered in two orientations: “in” (pointing towards the cavity) and “out” at a 2:1 population ratio. In their “out” arrangement, a disordered chloroform molecule was located within the calix cavity. Assuming that the preferred conformation in solution is similar to the crystal conformation, the observed eightfold symmetry observed in the NMR spectra can be ascribed to a fast dynamic process involving rotation about the carbonyl–aryl and methoxy–aryl bonds.

### 2.4. Demethylation of ***6b*** with Iodocyclohexane/DMF

An iodocyclohexane/DMF mixture was introduced by Wang, Duan and coworkers as a useful reagent for the demethylation of aryl methylethers [28]. This reagent has been successfully used in the exhaustive demethylation of calixarene methyl ether derivatives [29,30]. An attractive feature of the reagent is the slow release of HI generated by an elimination reaction. We therefore attempted the demethylation of **6b** by reaction with iodocyclohexane/DMF for 24, 48 and 96 h. Unfortunately, in all cases, the ^1^H NMR spectrum of the crude reaction mixture displayed a complex pattern of broad signals in both the *t*-Bu and aromatic regions, suggesting that the demethylation did not proceed to completion (Equation (2)).
(2)
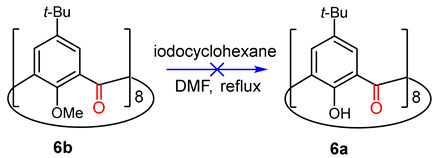


As an alternative pathway for the preparation of **6a**, we also examined the CrO_3_ oxidation of **3c** since if the oxidation of methylenes proceeds as intended, the readily hydrolyzed acetoxy group should provide synthetic access into the octahydroxy derivative.

### 2.5. Oxidation of Octaacetoxy p-tert-Butyl-calix[8]arene ***3c***

**Scheme 3:**
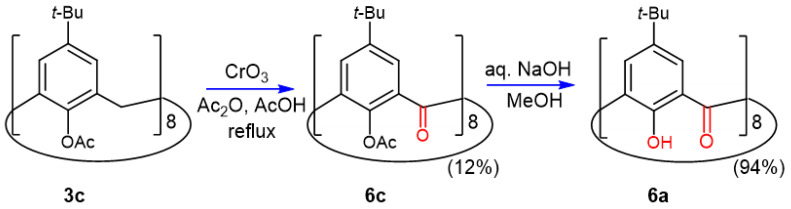
Preparation of octaacetoxyketocalix[8]arene **6c** and octahydroxyketocalix[8]arene **6a**.

We have previously shown that the oxidation of the 1,3-*alternate* form of tetracetoxycalix[4]arene **1c** to the corresponding tetraoxoketocalix[4]arene can be conducted by reaction with NBS in a CHCl_3_/H_2_O mixture under irradiation with a 100 W lamp. Although HBr is generated in the reaction, the acetate groups were not cleaved during the oxidation step [31]. However, preliminary experiments indicated that the attempted oxidation of **3c** with NBS under similar reaction conditions resulted only in decomposition products. We therefore resorted to Görmar’s reaction conditions (CrO_3_ in refluxing Ac_2_O/AcOH) [17]. Satisfactorily, the reaction afforded the desired octaacetoxyketocalix[8]arene **6c**, albeit in low yield (12%, Scheme 3).

**Figure 4:**
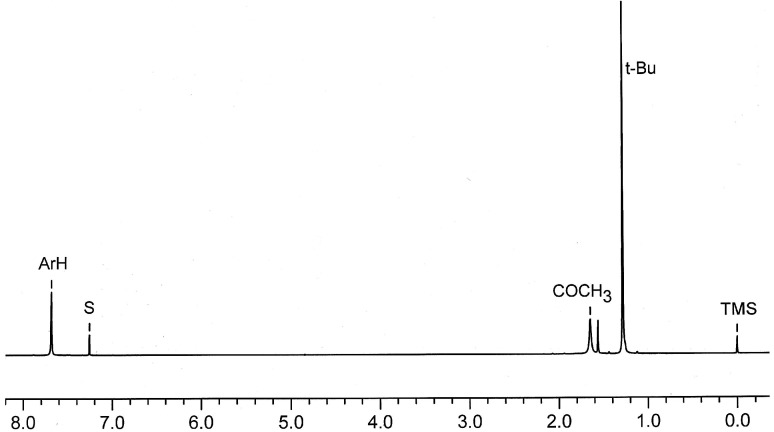
^1^H NMR spectrum (CDCl_3_, rt) of octaacetoxy ketocalix[8]arene **6c**.

The ^1^H NMR spectrum of **6c** displayed a simple spectrum with sharp singlets for the aromatic and *t*-Bu groups (Figure 4). As observed for **6b**, the aromatic signal was downfield shifted resonating at 7.69 ppm. Notably, the acetoxy group appeared as a broad signal at 1.65 ppm. The relative upfield shift of the acetoxy groups suggest that at least some of the acetoxy groups are under the shielding effects of the aromatic rings. The increased broadening of the acetoxy signal may be indicative of a dynamic exchange between conformers resulting in an observed time averaged signal. A similar broadening of the acetoxy signal in the ^1^H NMR spectrum was previously observed for the smaller analog **5c** [19].

### 2.6. Crystal Structure of Octaacetoxy Ketocalix[8]arene ***6c***

**Figure 5:**
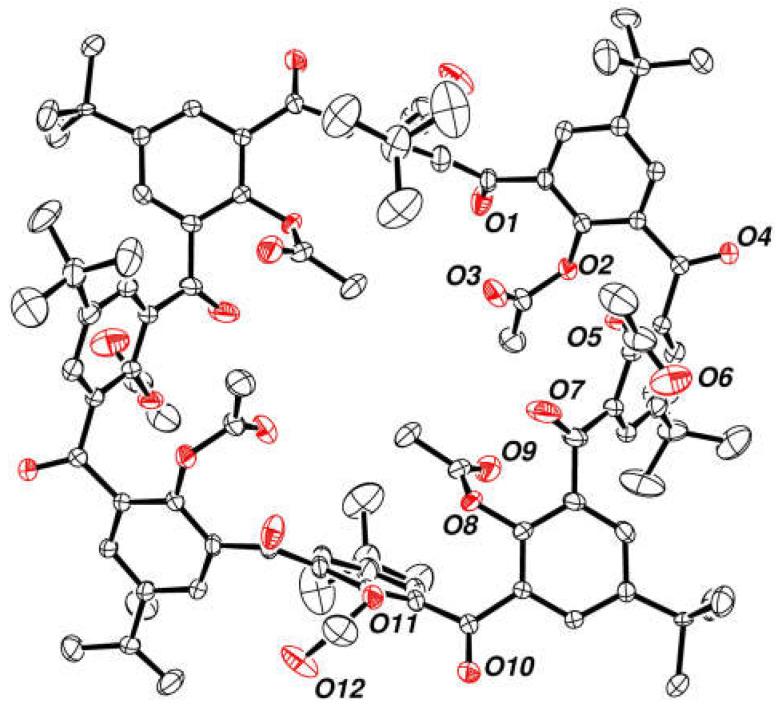
Crystal structure of octaacetoxyketocalix[8]arene **6c**. Disordered *t*-Bu groups and chlorobenzene molecule that cocrystallized are omitted for clarity.

A single crystal of **6c** was grown from chlorobenzene and submitted to X-ray crystallography. The solvent molecule that cocrystallized was disordered, and its hydrogen atoms were not located nor calculated. The molecule adopts in the crystal a conformation of *C*_i_ symmetry (Figure 5). The eight carbonyl groups can be divided into two types: “corner” and “edge”. The four “corner” carbonyls radiate from the center of the macrocycle. The acetoxy groups attached to four non-vicinal rings are oriented towards the cavity center, while the rest of the acetoxy groups are oriented “out”.

### 2.7. Preparation and Crystal Structure of Octahydroxyketocalix[8]arene ***6a***

The removal of the protecting acetyl groups of **6c** was conducted by reaction with aq. NaOH in MeOH yielding **6a** (Scheme 3). As with **6b** and **6c**, the ^1^H NMR spectrum of **6a** (500 MHz, CDCl_3_, rt) showed single singlets for all groups. The OH groups resonated at low field (δ 11.01 ppm), suggesting that these groups are involved in hydrogen bonds. A single crystal of **6a** was grown by the slow evaporation of an ethanolic solution and submitted to X-ray diffraction.

**Figure 6:**
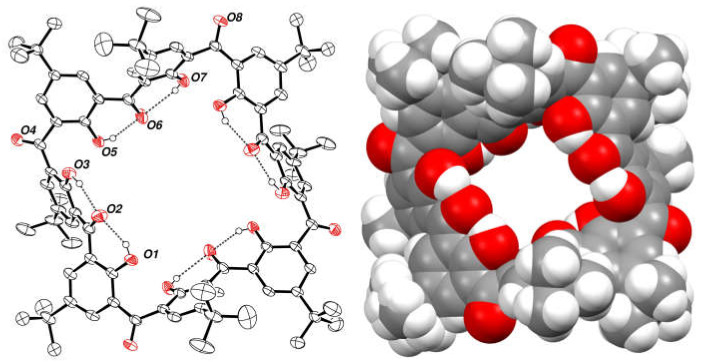
An ORTEP (**left**) and CPK representation (**right**) of the crystal structure of octahydroxy ketocalix[8]arene **6a**. Disordered *t*-Bu and ethanol molecules that cocrystallized are omitted. The molecule adopts a saddle-like conformation of idealized *S*_4_ symmetry. Four pairs or congruent hydrogen bonds are present, involving OH and carbonyl groups. Selected O…O distances (Å): O(1)O(2): 2.611(3), O(2)O(3): 2.584(3), O(5)O(6): 2.548(2), O(6)O(7): 2.696(2).

In contrast to **6b** and **6c**, **6a** adopts in the crystal a saddle-like conformation of crystallographic *C*_2_ symmetry and idealized *S*_4_ symmetry. In general, the parent **3a** displays a closed loop of hydrogen bonds where each OH serves both as a donor and acceptor of a hydrogen bond. A detailed analysis of the crystal structure of **3a** cocrystallized with different solvents showed that although in some cases, a solvent molecule can disrupt the closed loop, most of the intramolecular hydrogen bonds between neighboring phenol rings remain unaffected [32]. Remarkably, the hydrogen bond pattern in **6a** displays four pairs of convergent intramolecular hydrogen bonds involving carbonyl groups and a pair of geminal phenol rings (Figure 6). To the best of our knowledge, such a pattern is unprecedented for a calix[8]arene analog. The non-hydrogen-bonded carbonyls are located at the corners of the somewhat square-shaped geometry and are pointing away from the cavity center. An analysis of the crystal structures of substituted benzophenones in the literature has shown that in non-hydrogen-bonded derivatives, the C=O bond length is on the average 1.22 Å, whereas when hydrogen-bonded, this bond length increases to 1.24–1.25 Å [33]. In **6a,** the C=O bond lengths of the two nonequivalent carbonyls at the corners are O(8)C(44):1.221(3) and O(4)C(22):1.224(3) Å, whereas for the hydrogen-bonded carbonyls, they are O(6)C(33):1.247(3) and O(2)C(11):1.246(3) Å, in agreement with the previous analysis.

Pairs of convergent hydrogen bonds involving carbonyl oxygens as acceptors were not observed in the crystal structure of the smaller ketocalix[4]arene **4a**. It seems likely that the larger (and thus more flexible) macrocyclic scaffold in **6a** allows for conformations where pairs of OH groups on geminal rings are in steric proximity to a central carbonyl. These conformations are sterically inaccessible for the lower homolog **4a**.

**Figure 7:**
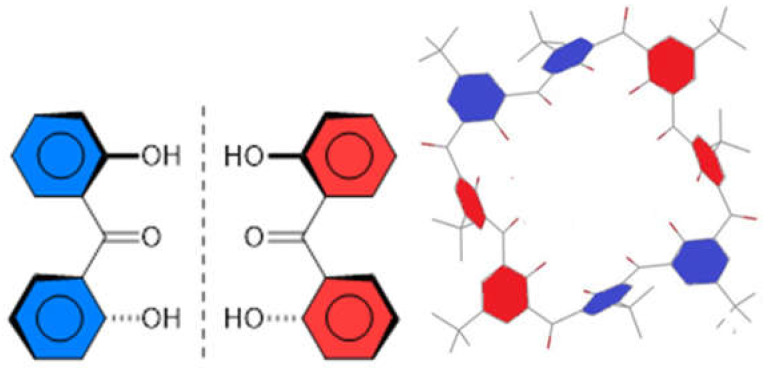
(**left**): The two possible helicities of a 2,2-dihydroxybenzophenone subunit possessing convergent hydrogen bonds. The two helicities are colored red and blue. (**right**): The helicities of the intramolecularly hydrogen bonded diarylketone subunits according to the color convention depicted on the left. The helicities alternate along the macrocycle resulting in a structure of idealized *S*_4_ symmetry.

The structure of **6a** can be viewed as comprising four non-overlapping diarylketone subunits. Diarylketones prefer a helical “propeller” conformation where both rings are twisted in the same direction relative to the C-C(=O)C central plane [33,34,35]. The propeller conformation is a compromise between two opposite effects: (i) to maximize the conjugation of the aryl rings with the carbonyls by attaining a coplanar arrangement and (ii) to minimize the steric repulsion between *ortho* hydrogens (or substituents if present) on both rings. This propeller conformation is chiral; thus, two enantiomeric arrangements are possible. These two arrangements are depicted in Figure 7. An inspection of the crystal structure of **6a** indicates that the two possible helicities alternate along the macrocycle, as expected for a conformation of idealized *S*_4_ symmetry (Figure 7).

### 2.8. Low-Temperature ^1^H NMR Spectra of ***6a***

**Figure 8:**
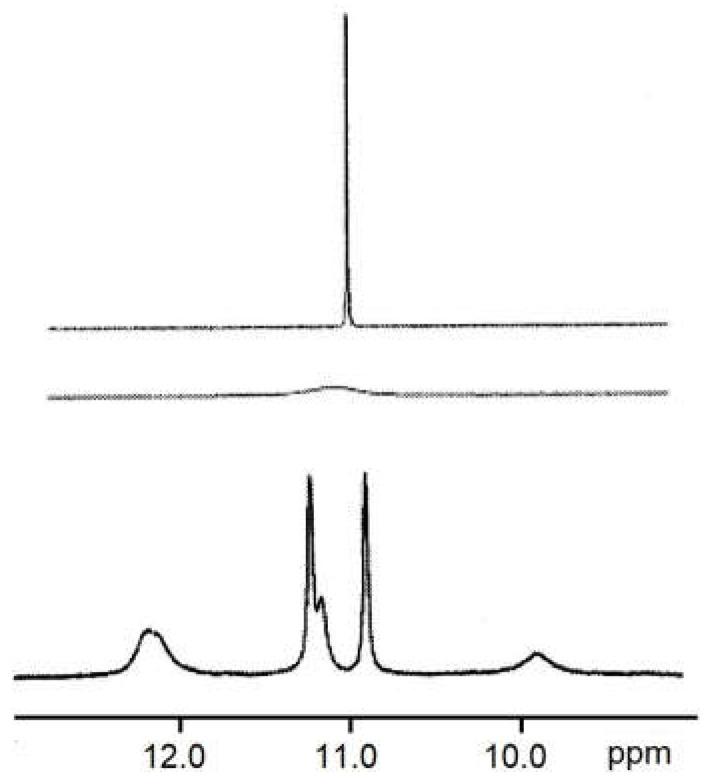
Temperature-dependent ^1^H NMR spectrum (400 MHz, CD_2_Cl_2_) of OH region of octahydroxy ketocalix[8]arene **6a**. From top to bottom: at 288, 217 and 188 K.

In the crystal conformation, the unique *C*_2_ axis is perpendicular to the main macrocyclic plane and exchanges pairs of opposite rings. However, this chiral conformation may be due to a minor distortion (due to crystal packing forces) of the more symmetric achiral conformation of *S*_4_ symmetry. In this conformation, alternating rings (i.e., in “1,3,5,7” or “2,4,6,8” positions) are exchanged by the *S*_4_ axis, but pairs of geminal rings are symmetry nonequivalent. Thus, it should be expected that if the conformation is “frozen” on the NMR timescale, precluding accidental isochrony, two OH signals should be observed. Upon lowering the temperature of an NMR sample in CD_2_Cl_2_, the OH signals broadened and decoalesced into two relatively sharp signals in a 1:1 ratio and three broad signals in a 1:1:2 ratio (Figure 8). If the two sharp singlets are assigned to a conformation of *S*_4_ symmetry, it can be concluded that an additional conformation with *C*_2_ or *C*_i_ symmetry is also present in solution.

## 3. Experimental Methods

### 3.1. General Information

All common reagents and solvents were used from commercial suppliers without further purification. ^1^H NMR and ^13^C NMR spectra were recorded at room temperature on DRX400 (Bruker, Karlsruhe, Germany) and AVANCE II 500 (Bruker, Karlsruhe, Germany) instruments. Proton chemical shifts (δ in ppm) were referenced to the residual protium resonance of the solvent (CDCl_3_, δ = 7.26 ppm) or to TMS. Carbon chemical shifts (δ in ppm) were referenced to the carbon resonances of the solvent (CDCl_3_, δ = 77.2 ppm). High-resolution mass spectra (HRMS) were recorded at the Analytical Chemistry Lab (The Hebrew University) using an X500R QTOF (SCIEX, Framingham, MA, USA) instrument. Melting points were measured using a Fisher-Johns (Fischer Scientific Co., Hampton, VA, USA) melting point apparatus and were uncorrected.

### 3.2. Crystal Data

CCDC (Deposition Number 2363603-2363605) contains the supplementary crystallographic data for this paper. These data can be obtained free of charge via https://www.ccdc.cam.ac.uk/structures/? (accessed on 28 August 2024) (or from the CCDC, 12 Union Road, Cambridge CB2 1EZ, UK; Fax: +44 1223 336033; E-mail: deposit@ccdc.cam.ac.uk).

**Crystal data for 6a**: empirical formula: C_98.5_H_108_O_21.25_, formula weight: 1631.84, temperature: 150.0(1) K, crystal system: monoclinic, space group: *I*2/a, *a*: 18.4709(5) Å, *b*: 22.0612(6) Å, *c*: 23.7204(7) Å, β: 90.484(3)°, *V*: 9665.5(5) Å^3^, *Z*: 4, ρcalc: 1.121 g/cm^3^, μ: 0.078 mm^−1^, F(000): 3476.0, crystal size: 0.24 × 0.09 × 0.08 mm^3^, radiation: Mo Kα (λ = 0.71073), 2θ range for data collection: 4.41 to 64.572°, index ranges: −23 ≤ h ≤ 27, −32 ≤ k ≤ 28, −33 ≤ l ≤ 35, reflections collected: 44,192, independent reflections: 14328 [R_int_ =0.0435, R_sigma_ =0.0584], data/restraints/parameters: 14,328/0/606, goodness-of-fit on F^2^: 1.028, final *R* indexes [I ≥ 2σ (I)]: *R*_1_: 0.0916, w*R*_2_: 0. 0.2518, final *R* indexes [all data]: *R*_1_: 0.1404, w*R*_2_: 0.2827, largest diff. peak/hole/e Å^−3^ 0.80/−0.34.

**Crystal data for 6b**: empirical formula: C_97.67_H_113.33_Cl_5_O_16_, formula weight: 1720.50, temperature: 200.0(1) K, crystal system: triclinic, space group: *P*-*1*, *a*: 12.4386(1)Å, *b*: 13.4767(2) Å, *c*: 14.6784(1) Å, α: 97.887(1)°, β: 93.365(1)°, γ: 96.941(1)°, *V*: 2412.32(4) Å^3^, *Z*: 1, ρcalc: 1.184 g/cm^3^, μ: 0.212 mm^−1^, F(000): 912.0, crystal size: 0.43 × 0.11 × 0.07 mm^3^, radiation: Mo Kα (λ = 0.71073), 2θ range for data collection: 4.174 to 64.604°, index ranges: −17 ≤ h ≤ 18, −19 ≤ k ≤ 19, −21 ≤ l ≤ 21, reflections collected: 104,881, independent reflections: 15145 [R_int_ = 0.0309, R_sigma_ = 0.0221], data/restraints/parameters: 15,145/0/631, goodness-of-fit on F^2^: 1.031, final *R* indexes [I ≥ 2σ (I)]: *R*_1_: 0.0695, w*R*_2_: 0.2071, final *R* indexes [all data]: *R*_1_: 0.0871, w*R*_2_: 0.2205, largest diff. peak/hole/e Å^−3^ 0.84/−0.66.

**Crystal data for 6c**: empirical formula: C_116_H_112_Cl_2_O_24_, formula weight: 1960.95, temperature: 150.0(1) K, crystal system: triclinic, space group: *P*-*1*, *a*: 10.0905(1) Å, *b*: 15.8706(2) Å, *c*: 17.1177(2) Å, α: 84.812(1)°, β: 76.830(1)°, γ: 82.254(1)°, *V*: 2639.74(5) Å^3^, *Z*: 1, ρcalc: 1.234 g/cm^3^, μ: 0.134 mm^−1^, F(000): 1034.0, crystal size: 0.26 × 0.19 × 0.09 mm^3^, radiation: Mo Kα (λ = 0.71073), 2θ range for data collection: 4.174 to 64.798°, index ranges: −14 ≤ h ≤ 14, −22 ≤ k ≤ 23, −23 ≤ l ≤ 24, reflections collected: 94,717, independent reflections: 16,346 [R_int_ = 0.0412, R_sigma_ = 0.0312], data/restraints/parameters: 16,346/0/778, goodness-of-fit on F^2^: 1.027, final *R* indexes [I ≥ 2σ (I)]: *R*_1_: 0.0737, w*R*_2_: 0.1987, final *R* indexes [all data]: *R*_1_: 0.0918, w*R*_2_: 0.2101, largest diff. peak/hole/e Å^−3^ 0.57/−0.68.

### 3.3. Synthesis of Compounds ***6a***–***6c***

#### 3.3.1. Preparation of 5,11,17,23,29,35,41,47-Octa-*tert*-butyl-49,50,51,52,53,54,55,56-octahydroxy-2,8,14,20,26,32,38,44-octaoxo-calix[8]arene (**6a**)

A mixture of **6c** (0.5 g, 0.29 mmol), aq. NaOH (75 mL, 2 M) and methanol (50 mL) was heated to reflux for 4 h. After cooling to rt, the mixture was acidified with conc. HCl until pH = 1, and the solid was filtered by vacuum yielding 0.48 g of a yellow solid. Trituration with cold methanol afforded 0.38 g **6a** (94%), mp 305–307 °C.

IR ν = 3527 (OH), 1669 (C=O) cm^−1^. ^1^H NMR (CDCl_3_, 500 MHz) δ 11.01 (s, 8H, OH), 7.86 (s, 16H, ArH), 1.35 (s, 72H, *t*-Bu) ppm. ^13^C NMR (CDCl_3_, 125 MHz) δ 198.4, 158.9, 141.6, 133.1, 124.8, 34.5, 31.3 ppm. HRMS (ESI-QTOF) *m*/*z* calcd. for C_88_H_96_O_16_ + H^+^: 1409.6771. Found: 1409.6730.

#### 3.3.2. Preparation of 5,11,17,23,29,35,41,47-Octa-*tert*-butyl-49,50,51,52,53,54,55,56-octamethoxy-2,8,14,20,26,32,38,44-octaoxocalix[8]arene (**6b**)

A mixture of octamethoxycalix[8]arene **3b** (4.7 g, 4.36 mmol), NBS (19.7 g, 110.0 mmol), chloroform (90 mL) and water (2 mL) was refluxed during 3.5 h while irradiated with a spotlight (100 W). After cooling, the solution was washed successively with aq Na_2_S_2_O_5_, water, aq NaHCO_3_, water, dried over MgSO_4_ and evaporated. A ^1^H NMR analysis of the crude product indicated a nearly complete oxidation of the methylene groups. The crude product was recrystallized from CHCl_3_/EtOH, yielding the product (1.82 g, 36% yield), mp 312 °C.

^1^H NMR (CDCl_3_, 400 MHz) δ 7.60 (s, 16H, ArH), 3.23 (s, 24H, OMe), 1.25 (s, 72H, *t*-Bu) ppm. ^13^C NMR (CDCl_3_, 100 MHz) δ 195.3, 155.9, 145.9, 133.3, 130.6, 63.1, 34.5, 31.1 ppm. HRMS (ESI-QTOF) *m*/*z* calcd. for C_96_H_112_O_16_ + H^+^: 1521.8023. Found: 1521.7952.

#### 3.3.3. Preparation of 5,11,17,23,29,35,41,47-Octa-*tert*-butyl-49,50,51,52,53,54,55,56-octaacetoxy-2,8,14,20,26,32,38,44-octaoxo-calix[8]arene (**6c**)

To a mixture of octaacetoxycalix[8]arene **3c** (3.03 g, 1.85 mmol), 200 mL acetic anhydride and 4 mL acetic acid was added CrO_3_ (6.5 g, 0.065 mmol). The mixture was heated to reflux for 3h. During the heating period, the color of the reaction changed from dark orange to green. After cooling to rt, the mixture was filtered through a sintered glass filter, 200 mL of chloroform was added and the mixture was washed with water (200 mL) several times until the water phase was no longer colored. The organic phase was dried (MgSO_4_) and evaporated. The residue was recrystallized from CHCl_3_/MeOH, yielding 0.4 g (12%) **6c**, mp 310–312 °C.

^1^H NMR (CDCl_3_, 500 MHz) δ 7.69 (s, 16H, ArH), 1.65 (br s, 24H, CH_3_C=O), 1.28 (s, 72H, *t*-Bu) ppm. ^13^C NMR (CDCl_3_, 125 MHz) δ 192.3, 168.5, 149.1, 144.9, 132.4, 130.8, 34.9, 31.0, 20.0 ppm. Anal. calcd. for C_104_H_112_O_24_: C, 71.54, H, 6.47. Found: C, 71.54, H, 6.46. HRMS (ESI-QTOF) *m*/*z* calcd. for C_104_H_112_O_24_ + H^+^: 1745.7616. Found: 1745.7603.

## 4. Conclusions

Octamethoxy and octaacetoxyketocalix[8]arenes can be prepared by the oxidation of the corresponding calix[8]arenes with NBS/light and CrO_3_, respectively. An X-ray analysis of a crystal of octahydroxyketocalix[8]arene **6a** grown from ethanol indicates that the molecule adopts a conformation of idealized *S*_4_ symmetry where the helicities of the dihydroxybenzophenone subunits alternate along the macrocycle. The carbonyl groups disrupt all the intramolecular hydrogen bonds between phenol rings.

## Data Availability

The original contributions presented in this study are included in this article/Supplementary Material; further inquiries can be directed to the corresponding author.

## Supplementary Materials

The following supporting information can be downloaded at https://www.mdpi.com/article/10.3390/molecules29174094/s1, Figure S1: ^1^H NMR spectrum (500 MHz, CDCl_3_, rt) of **6a,** Figure S2: ^13^C NMR spectrum (125 MHz, CDCl_3_, rt) of **6a,** Figure S3: HRMS of **6a**, Figure S4: ^1^H NMR spectrum (400 MHz, CDCl_3_, rt) of **6b**, Figure S5: ^13^C NMR spectrum (100 MHz, CDCl_3_, rt) of **6b**, Figure S6: HRMS of **6b**, Figure S7: **^1^**H NMR spectrum (500 MHz, CDCl_3_, rt) of **6c**, Figure S8: ^13^C NMR spectrum (125 MHz, CDCl_3_, rt) of **6c**, Figure S9: HRMS of **6c**.
