# Supplementary material for: The Preparation and Crystal Structures of Octaoxoketocalix[8]arene Derivatives: The Ketocalixarene Counterparts of the Largest “Major” Calixarene
Source: Molecules. 2024 Aug 29;29(17):4094. doi: 10.3390/molecules29174094 (PMC11397158; doi:10.3390/molecules29174094)

Supplementary Material

Preparation and Crystal Structures of Octaoxoketocalix[8]arene Derivatives: The Ketocalixarene Counterparts of the Largest "Major" Calixarene

Katerina Kogan, Suheir Omar, Benny Bogoslavsky and Silvio E. Biali*

Institute of Chemistry, The Hebrew University of Jerusalem,

Jerusalem 9190401, Israel.

Email: silvio.biali@mail.huji.ac.il

Figure S1. ^{1}H NMR spectrum (500 MHz, $CDCl_3$, rt) of **6a**

Figure S2. ^{13}C NMR spectrum (125 MHz, $CDCl_3$, rt) of **6a**

Figure S3. HRMS of **6a**

Figure S4. ^{1}H NMR spectrum (400 MHz, $CDCl_3$, rt) of **6b**

Figure S5. ^{13}C NMR spectrum (100 MHz, $CDCl_3$, rt) of **6b**

Figure S6. HRMS of **6b**

Figure S7, ^{1}H NMR spectrum (500 MHz, $CDCl_3$, rt) of **6c**

Figure S8. ^{13}C NMR spectrum (125 MHz, $CDCl_3$, rt) of **6c**

Figure S9. HRMS of **6c**

Figure S1. ^{1}H NMR spectrum (500 MHz, $CDCl_3$, rt) of **6a**

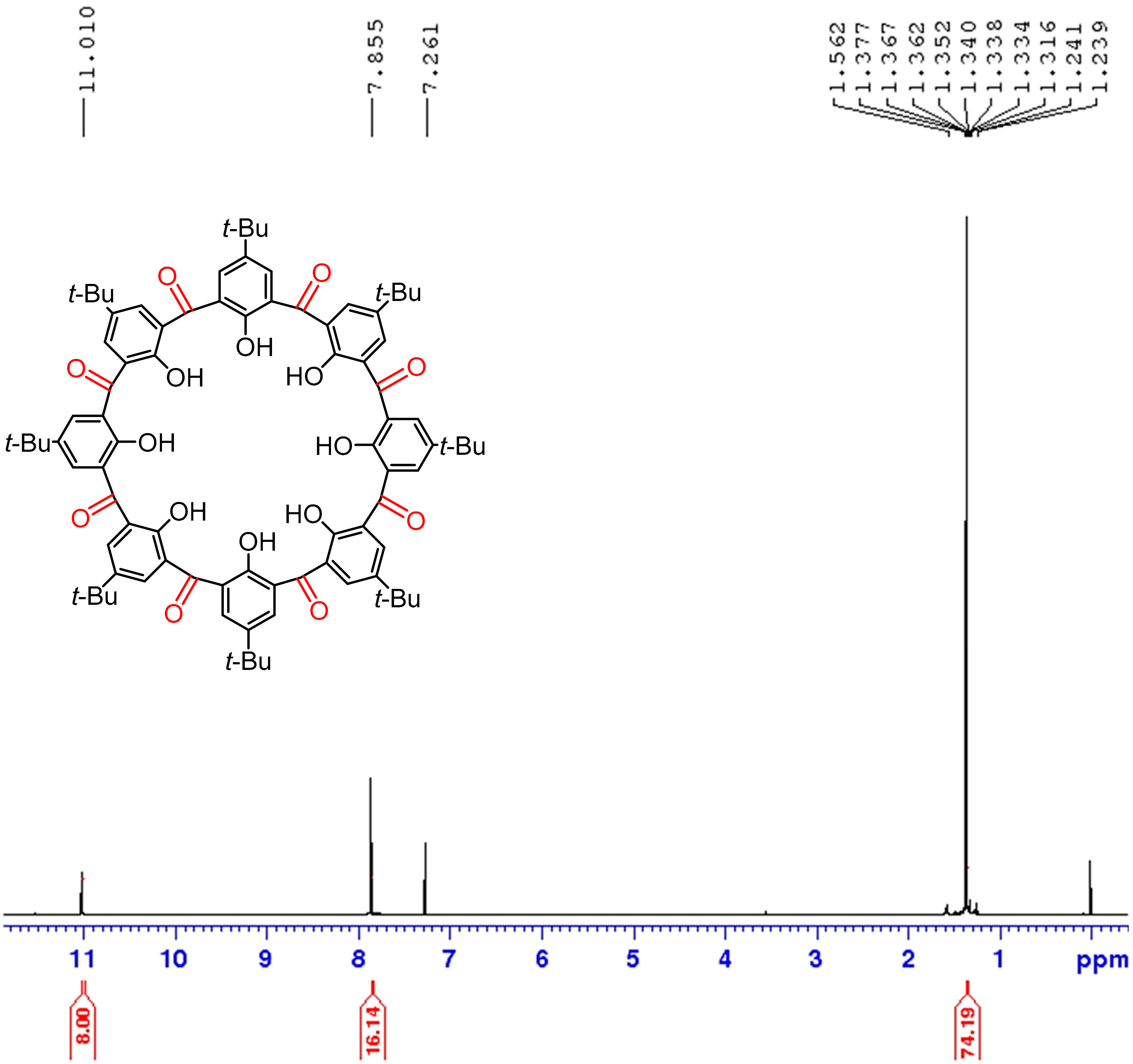

Figure S2. ^{13}C NMR spectrum (125 MHz, $CDCl_3$, rt) of **6a**

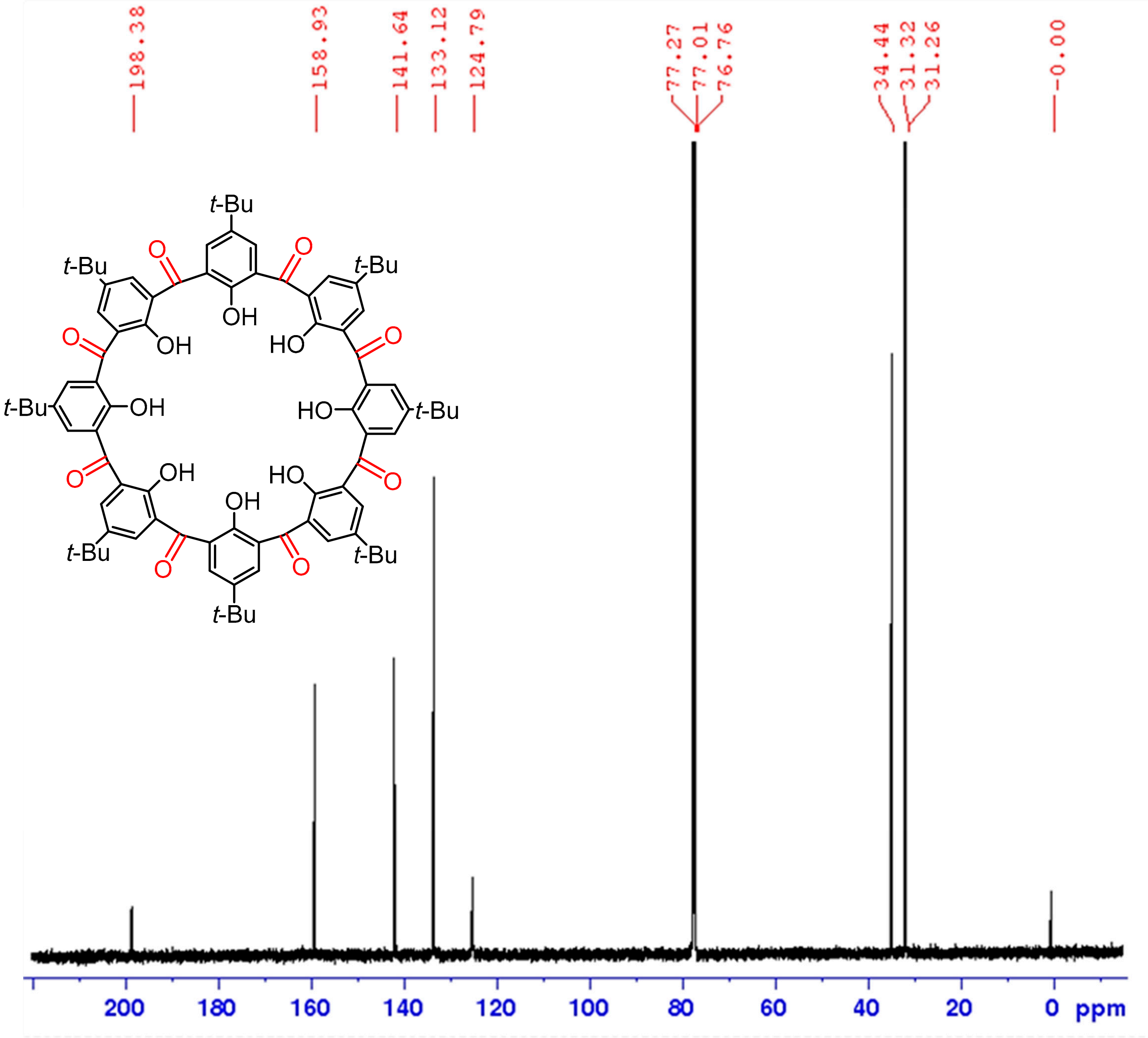

Figure S3. HRMS of **6a**

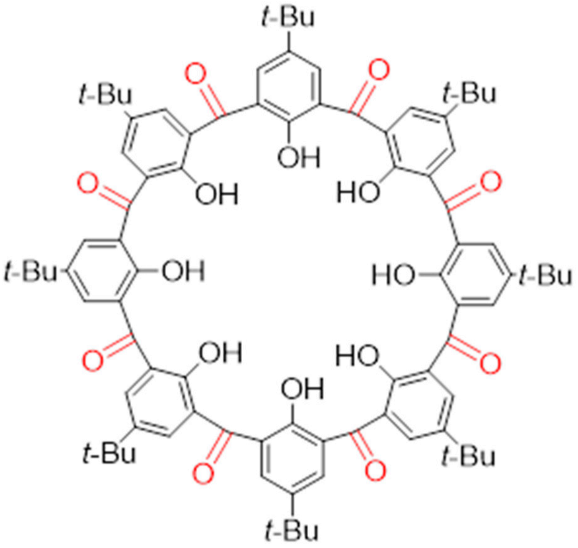

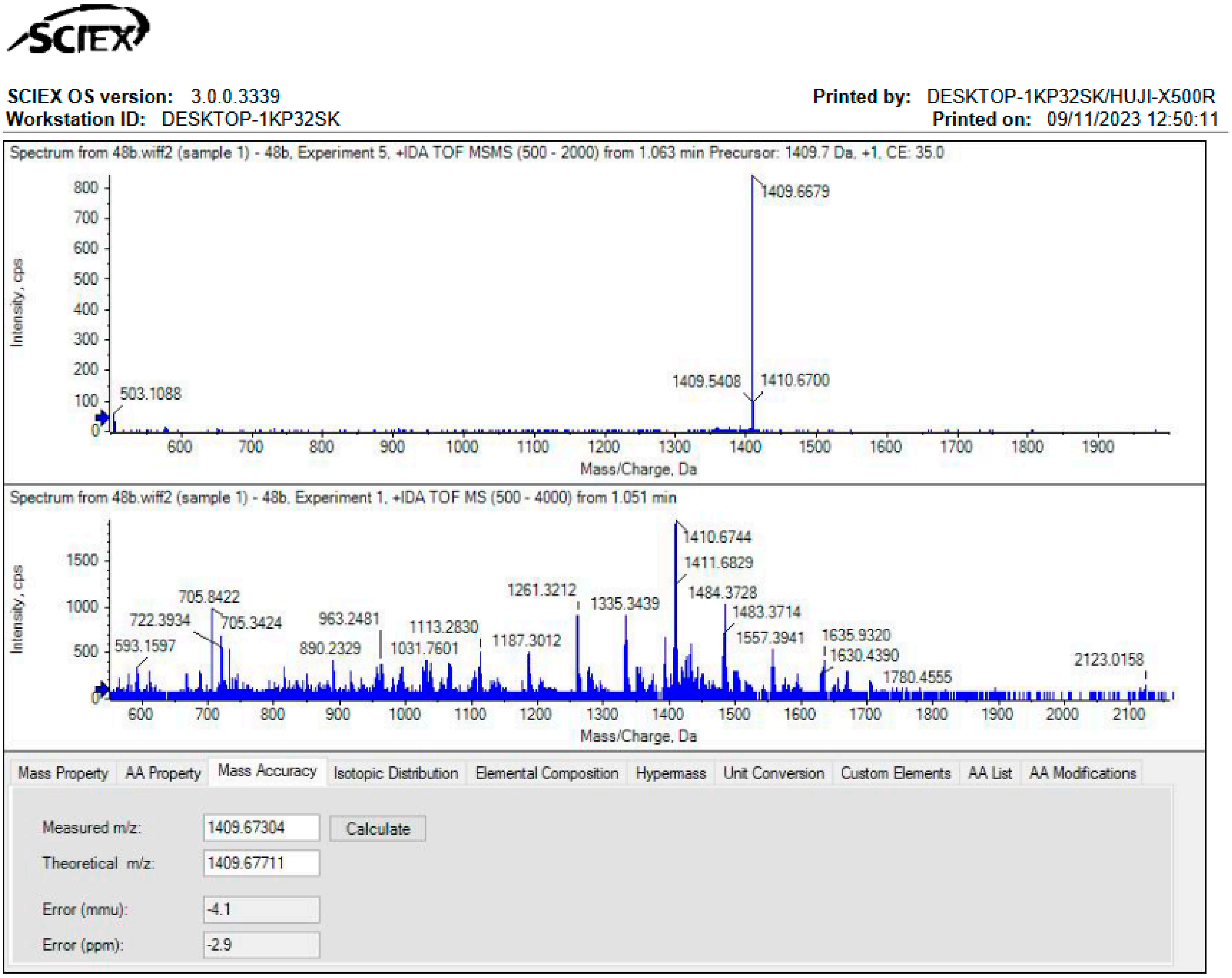

Figure S4. ^{1}H NMR spectrum (500 MHz, $CDCl_3$, rt) of **6b.**

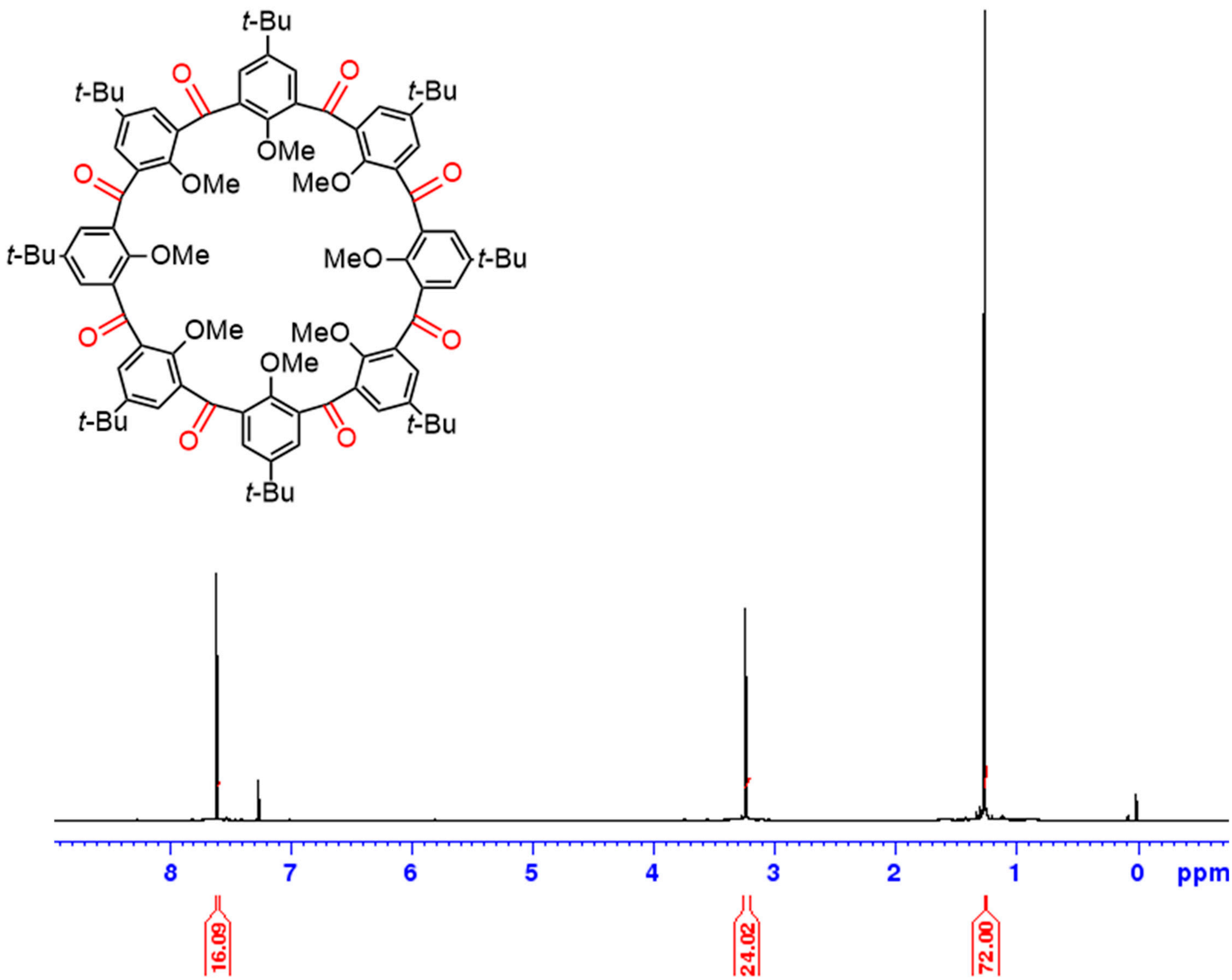

Figure S5. ^{13}C NMR spectrum (125 MHz, $CDCl_3$, rt) of **6b.**

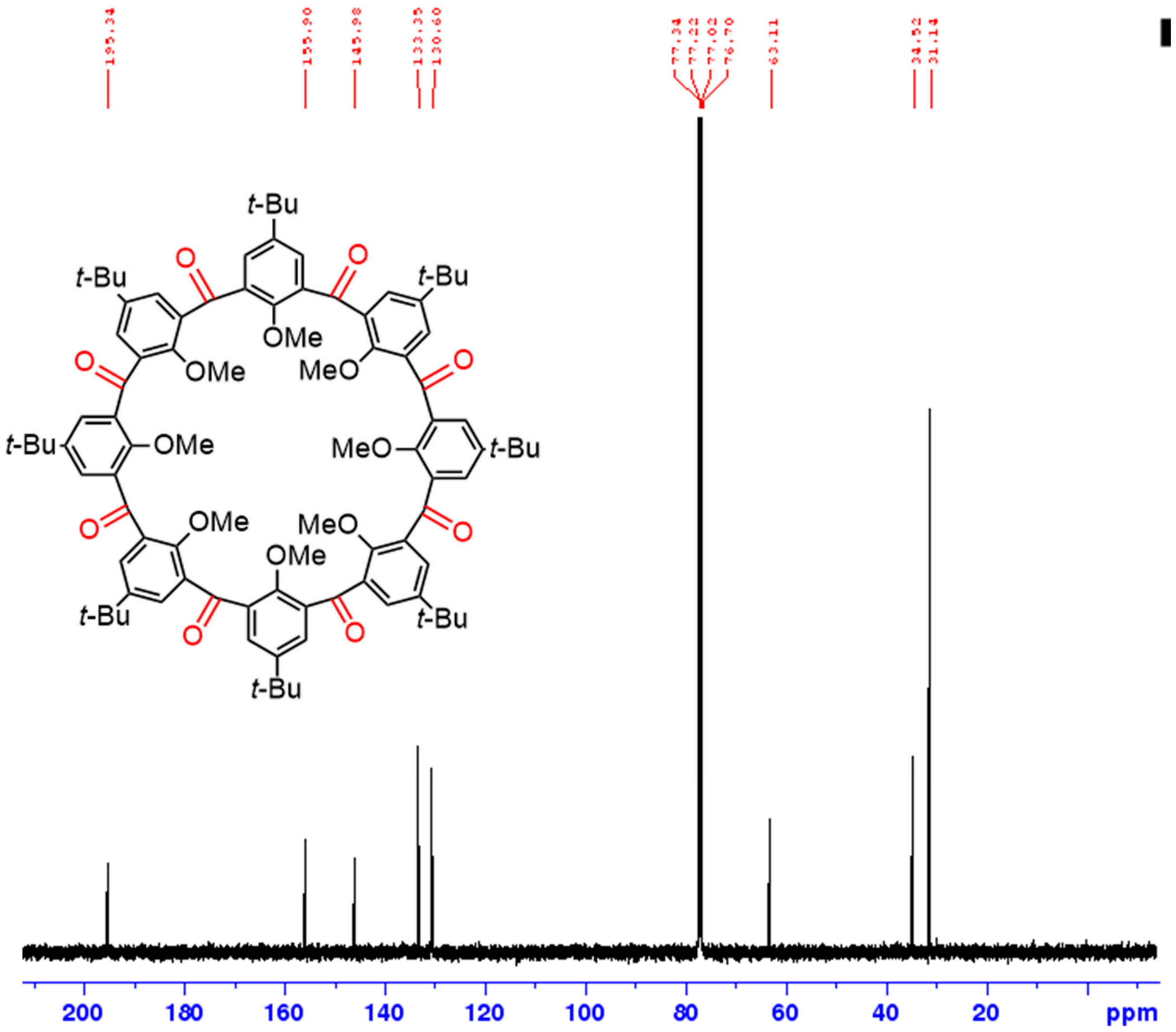

Figure S6. HRMS of **6b**

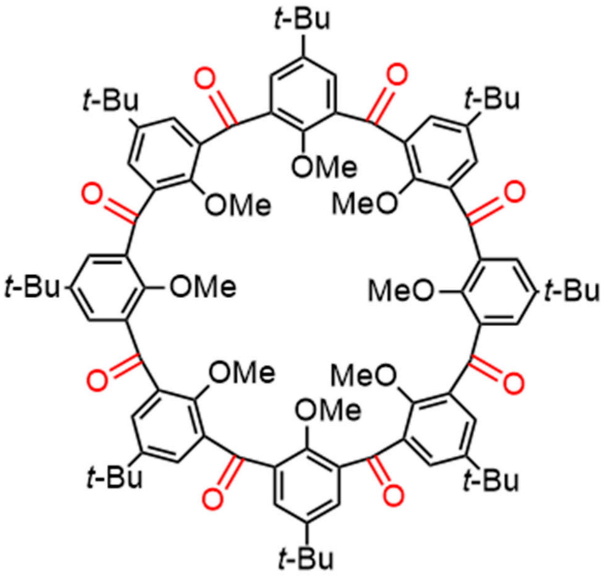

SCIEX OS version: 3.0.0.3339
Workstation ID: DESKTOP-1KP32SK
Printed by: DESKTOP-1KP32SK/HUJI-X500R
Printed on: 22/05/2024 15:14:13

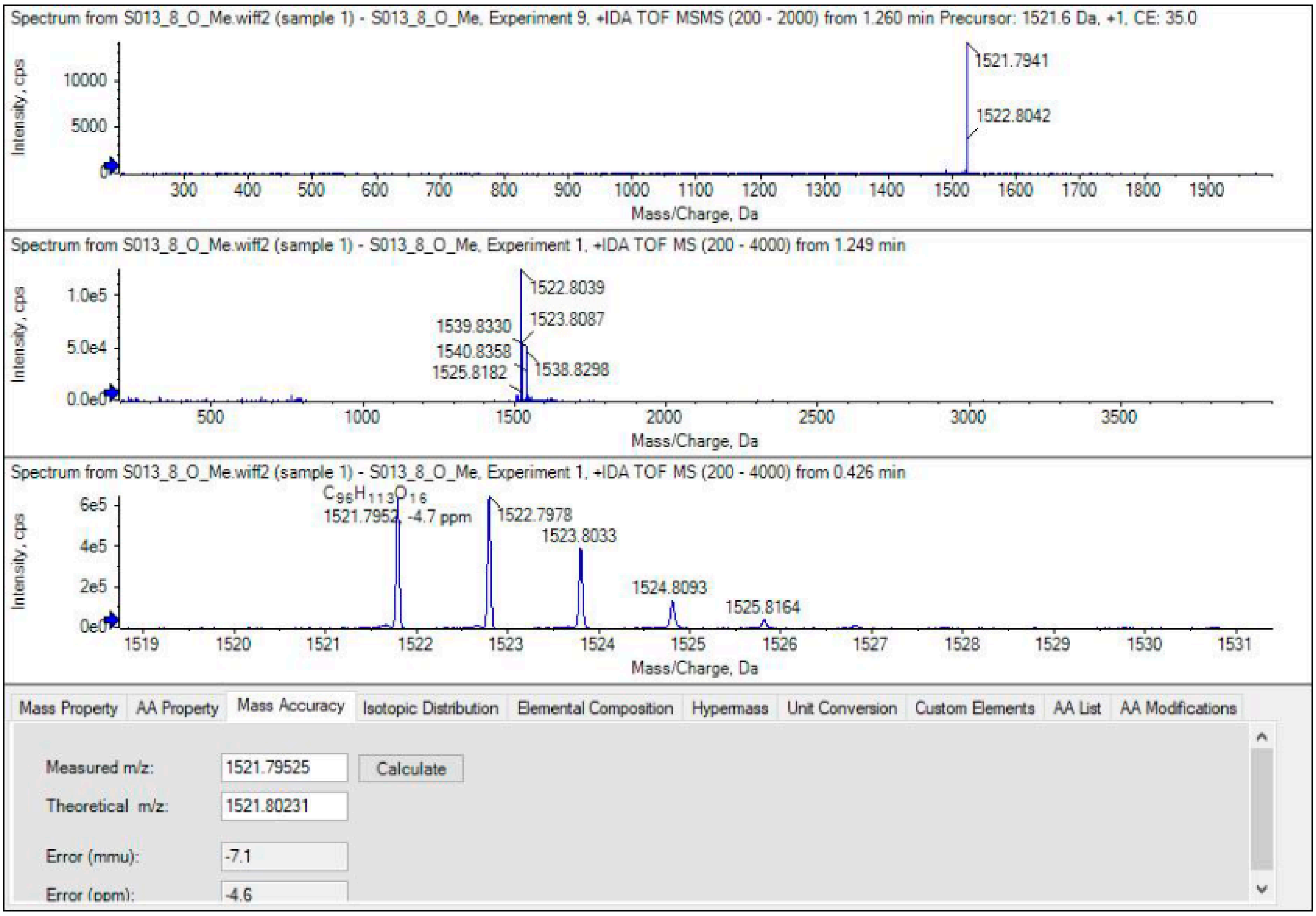

Figure S7. ^{1}H NMR spectrum (500 MHz, $CDCl_3$, rt) of **6c**

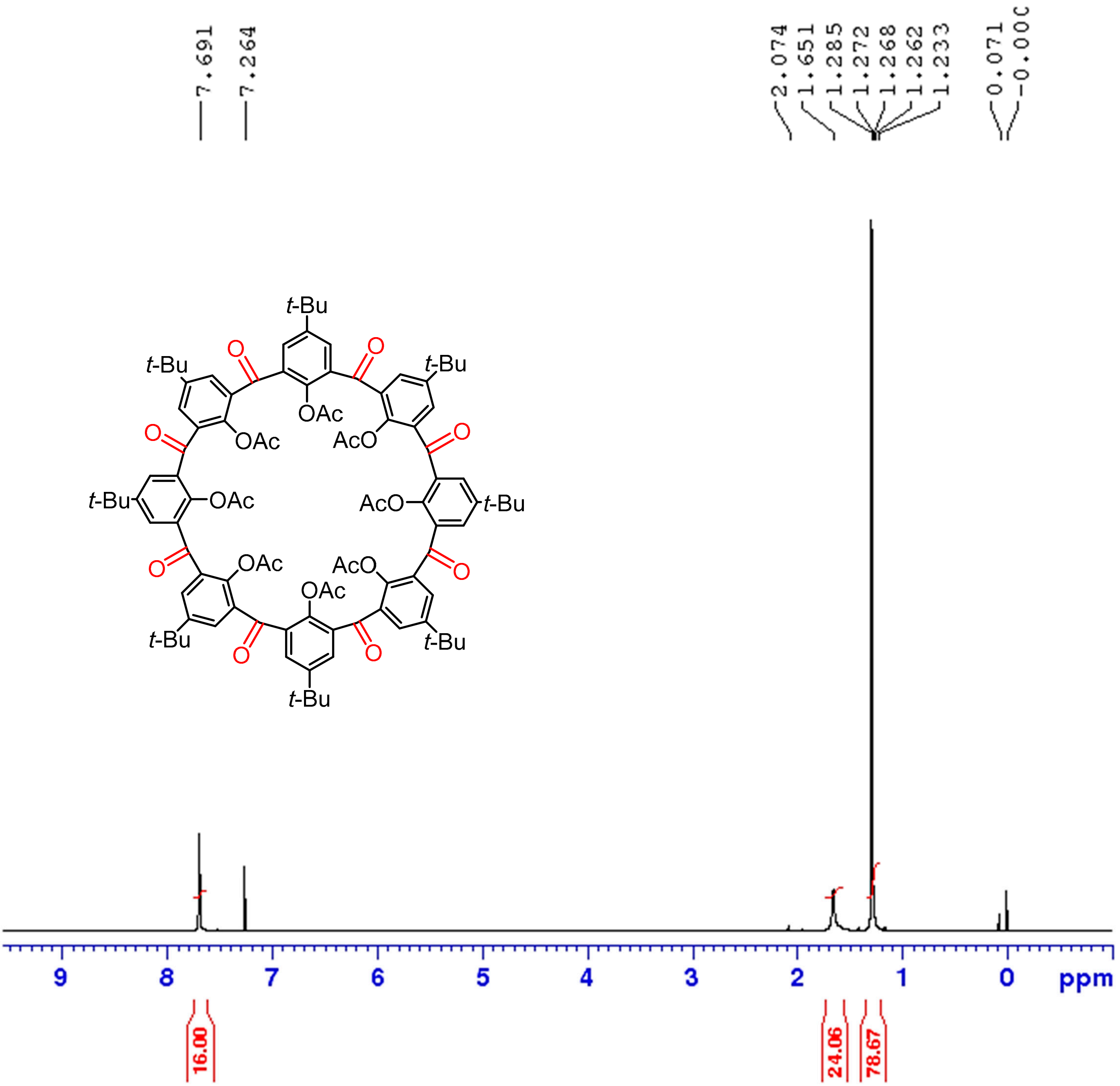

Figure S8. ^{13}C NMR spectrum (125 MHz, $CDCl_3$, rt) of **6c**

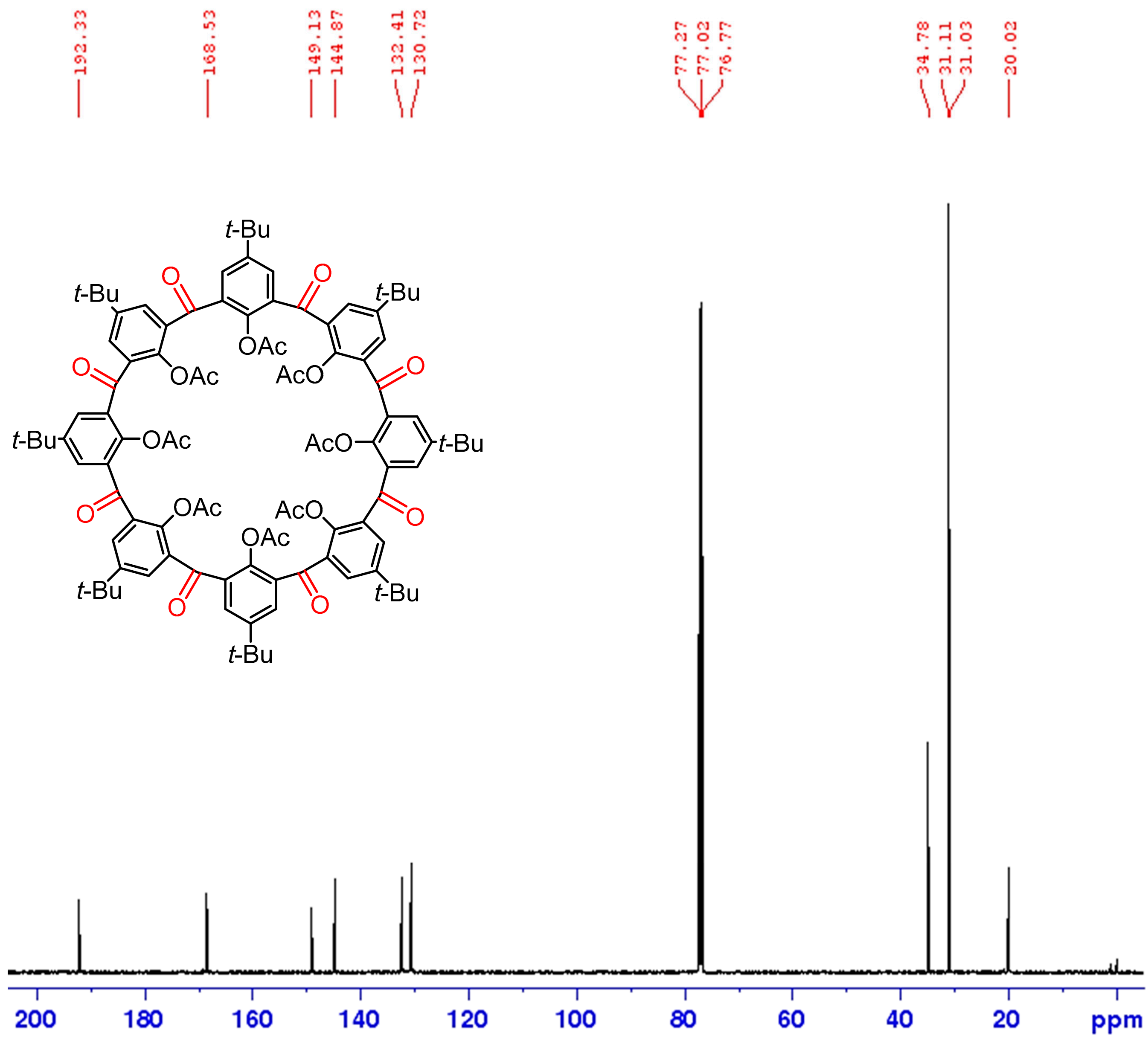

Figure S9. HRMS of **6c**

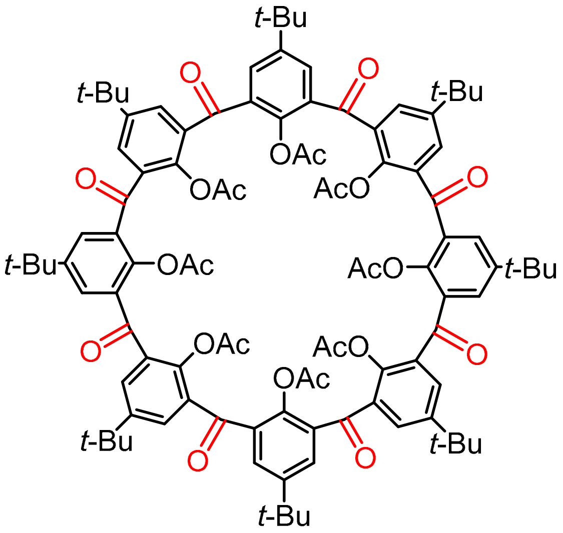

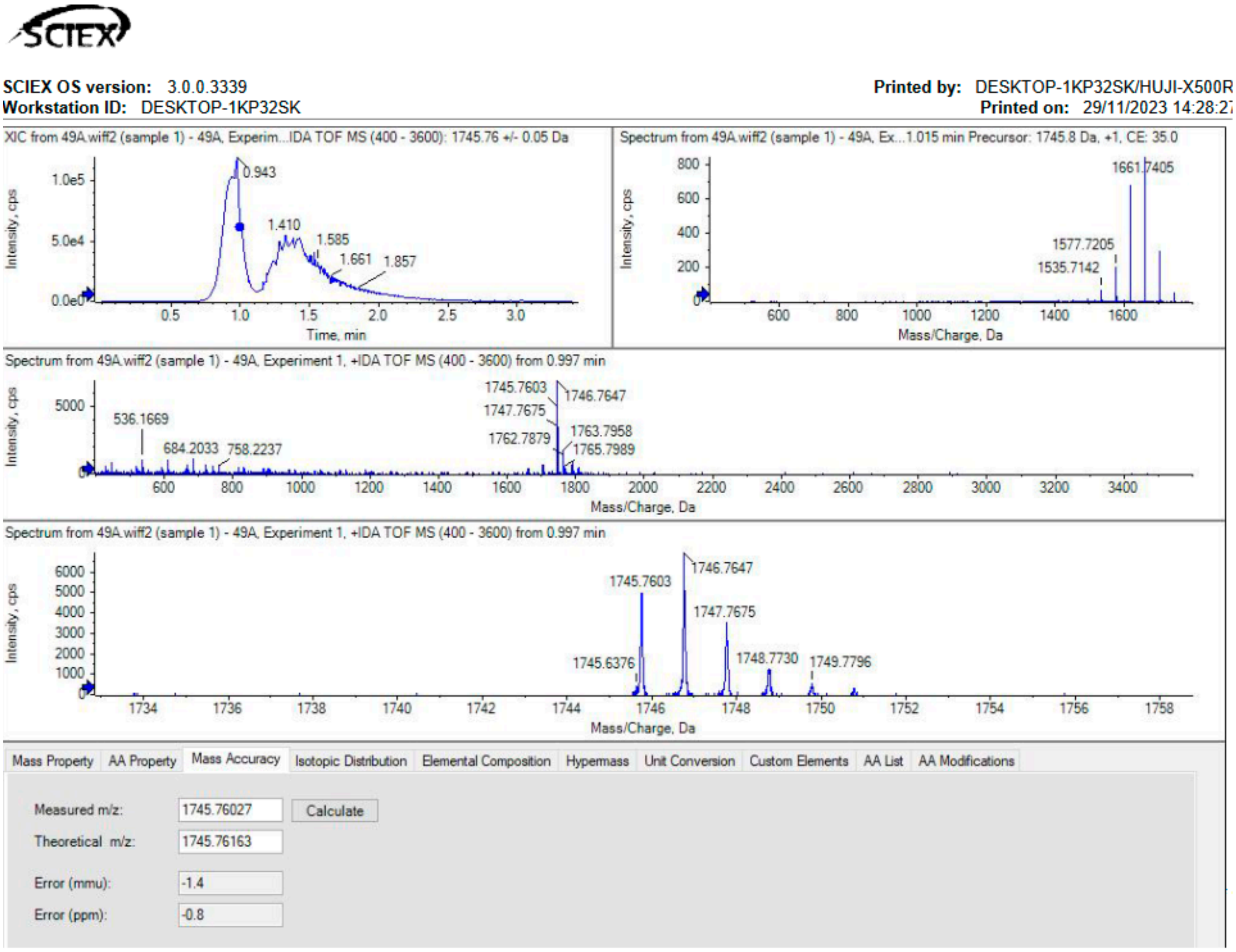

Supplement: Supplementary file 1 [file molecules-29-04094-s001.zip › molecules-3115698-supplementary.pdf]
